# The host protein cyclophilin A restricts nuclear entry of HIV-1 mutants by reducing the elasticity of the viral capsid

**DOI:** 10.1371/journal.ppat.1013910

**Published:** 2026-01-29

**Authors:** Jun Hong, Akshay Deshpande, Yatish Thakare, Lora Simonovsky, AidanDarian W. Douglas, Conall Mc Guinness, Noa Rotem-Dai, Michelle L. Kortyna, J. Ole Klarhof, Jiong Shi, Leo C. James, Till Boecking, Ashwanth C. Francis, Itay Rousso, Christopher Aiken

**Affiliations:** 1 Vanderbilt University Medical Center, Department of Pathology, Microbiology and Immunology and Vanderbilt Institute for Infection, Immunology, and Inflammation, Nashville, Tennessee, United States of America; 2 Ben-Gurion University of the Negev, Department of Physiology and Cell Biology, Beersheva, Israel; 3 Department of Biological Sciences and Institute of Molecular Biophysics, Florida State University, Tallahassee, Florida, United States of America; 4 EMBL Australia Node in Single Molecule Science, School of Biomedical Sciences, UNSW, Sydney, Australia; 5 MRC Laboratory of Molecular Biology, Cambridge, United Kingdom; University of Wisconsin, UNITED STATES OF AMERICA

## Abstract

Binding of the host protein cyclophilin A (CypA) to the viral capsid exerts multiple effects on HIV-1 infection, including enhancement of reverse transcription, stabilization of the capsid, and promotion of nuclear entry. CypA can also inhibit infection of selected HIV-1 mutants by a poorly understood mechanism. Using atomic force microscopy methods, we previously showed that HIV-1 cores are highly elastic and that mutants with reduced capsid elasticity are impaired for nuclear entry and infection of nondividing cells. Here we demonstrate that binding of CypA to the capsids of such mutants inhibits their nuclear entry by further reducing the elasticity of their capsids. These effects were reversed by suppressor mutations that restored elasticity to the mutant capsids. Our results define the mechanism by which CypA controls HIV-1 nuclear entry. We hypothesize that nuclear entry involves temporal modulation of capsid elasticity by host proteins prior to and during passage through the nuclear pore.

## Author summary

In this study, we used advanced atomic force microscopy to show that the binding of the host cell protein cyclophilin A (CypA) to the HIV-1 capsid reduces its elasticity—a property required for HIV-1 nuclear entry. The results reveal a new effect of a capsid-binding host protein during infection, and they help explain the impaired ability of various HIV-1 mutants to enter the nucleus to infect cells. Combined with our previous discovery that the elasticity of the viral capsid is important for HIV-1 nuclear entry, the results suggest that other host proteins may promote or inhibit HIV-1 infection by controlling the elasticity of the HIV-1 capsid during nuclear entry. The results may also help explain how the efficacy of capsid-targeting drugs is enhanced by CypA.

## Introduction

An obligate step in infection by retroviruses is integration, which requires access to host cell chromatin residing within the nucleus. As a lentivirus, HIV-1 can enter the nuclei of nondividing cells, including resting T cells and terminally differentiated macrophages. This ability is endowed by the viral capsid [1,2], a closed lattice of hexamers and pentamers forming a shell encasing the viral genome and enzymes required for synthesis and integration of proviral DNA. During early stages of infection, the capsid promotes viral reverse transcription by sequestering the internal components of the viral core including reverse transcriptase, the nucleocapsid protein, and the viral RNA genome [3–6]. The capsid also interacts with components of the nuclear pore complex (NPC) and passes through it while remaining mostly or fully intact [7–9]. While the detailed mechanism by which traversal of the NPC by the megadalton-sized viral core occurs is unknown, recent evidence indicates that the capsid serves as a nuclear transport receptor by forming polyvalent interactions with FG repeat domains of several nucleoporins which form a gel-like plug in the central channel of the NPC [10,11]. Thus, by mimicking nuclear import receptors, the capsid may penetrate the plug without employing host cell transport factors required for cellular cargo.

In addition to nucleoporin binding, nuclear entry of the HIV-1 core depends on a specific physical property of the viral capsid. Using atomic force microscopy (AFM) to examine the consequences of strong compression of native HIV-1 cores, we recently demonstrated that the HIV-1 capsid is highly elastic and that this property is tightly linked to efficient nuclear entry [12]. In that study, amino acid substitutions in CA that were found to inhibit nuclear entry also reduced the elasticity (i.e., increased the brittleness) of the purified mutant HIV-1 cores. HIV-1 pseudorevertants that emerged during cell culture adaptation of two such mutants restored elasticity, linking elasticity to HIV-1 infection of nondividing cells. We also observed that the capsid-targeting inhibitors PF74 and Lenacapavir reduced HIV-1 capsid elasticity, further linking nuclear entry to elasticity.

Interactions of the HIV-1 capsid with host proteins other than nucleoporins may also affect nuclear penetration of the viral core. One such protein, cyclophilin A (CypA), binds to a flexible loop on the surface of the capsid protein (CA). The effects of CypA on HIV-1 infection are numerous and varied (reviewed in [13]). Treatment of target cells with the inhibitor cyclosporin A (CsA) prevents CypA from binding to the incoming viral capsid, preventing infection by inhibiting reverse transcription, nuclear entry, and possibly integration, depending on the cell type used in the experiment. In early studies, selection for resistance to CsA resulted in the identification of two CA substitutions (A92E and G94D) that independently conferred resistance of HIV-1 to CsA [14,15]. Subsequently, an additional substitution (T54A) in a distinct region of CA was shown to exhibit a similar phenotype [16]. These mutations do not prevent CypA binding to the capsid, which can be blocked by other substitutions (G89V, P90A) in CA. Further adaptation of CsA-dependent mutants resulted in acquisition of a suppressor mutation encoding the A105T substitution, which restored the ability of the A92E, G94D, and T54A mutants to infect cells in the absence of CsA [17]. Thus, the resulting mutant viruses are neither inhibited by CsA nor do they require it for infection.

The mechanism by which binding of CypA to the HIV-1 capsid inhibits infection by some CA mutants is unknown. CypA binding can stabilize the HIV-1 capsid and increase its stiffness, which is a physical property determined by the applied force required to reversibly deform the surface of an object under slight compression [18,19]. However, neither capsid stabilization nor stiffness alteration has been linked to CypA inhibition of infection. In this study, we tested the hypothesis that CypA binding to the viral capsid reduces its elasticity, thereby inhibiting nuclear entry. We observed that CypA reduced the elasticity of wild type HIV-1 cores in vitro in a concentration-dependent manner. HIV-1 mutants with intrinsically inelastic capsids were rendered even more brittle by addition of CypA. Ablation of CypA binding to the viral capsid in target cells restored the nuclear entry of HIV-1 mutants with inelastic capsids. Moreover, the A92E mutant, which is inhibited for infection of nondividing cells by CypA, was rendered brittle upon addition of CypA in vitro, and this effect was abolished by addition of the A105T suppressor. We also found that A105T enhanced the infectivity of the E45A mutant, particularly in nondividing cells, and addition of the E45A suppressor mutation R132T to A92E and T54A mutants rendered them able to infect nondividing cells. Collectively, our results support the hypothesis that CypA inhibits nuclear entry of some HIV-1 mutants by further reducing the elasticity of their capsids, as reflected in increased breakage upon compression.

## Results

### Infection of nondividing cells by HIV-1 mutants with inelastic capsids is restored by inhibition of CypA

**Fig 1:**
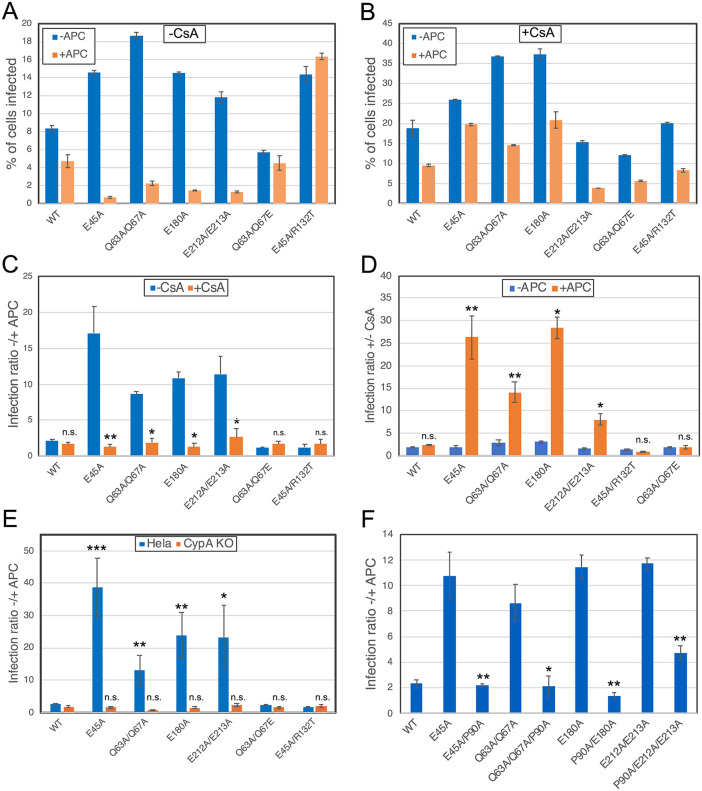
Binding of CypA to the viral capsid inhibits HIV-1 infection of nondividing cells by HIV-1 mutants with inelastic cores. Infection of wild type (A-E, F) and CypA KO Hela (E) cells with HIV-1 reporter viruses expressing GFP was quantified by flow cytometry. Cells were seeded in the presence and absence of aphidicolin (APC) to induce cell cycle arrest. In panel B, cells were infected in the presence of the CypA inhibitor CsA. Graphs in A and B show results from one representative of three experiments, with average values from duplicate infections. Error bars show the range of the two values. Panel C shows the infection ratios for dividing vs. nondividing cells in the presence and absence of CsA. Panel D shows the infection ratios with and without CsA in dividing and arrested cells from the same results shown in panel **C.** Mean values from four independent experiments are shown, with error bars representing standard deviations. Symbols represent results of statistical analysis of the effect of CsA (panel C) and cell cycle arrest (panel D) for each virus. *: p < 0.05; **: p < 0.01; ***: p < 0.001; n.s.: not significant. Panel E shows results of cell cycle arrest on infection of wild type and CypA KO cells. Statistical symbols represent comparisons between values from mutant and wild type viruses. Results shown in panel F show the effects of cell cycle arrest on infection by viruses containing the P90A substitution that prevents binding of CypA to the viral core in target cells. Values shown are means from three independent experiments, with error bars representing standard deviations. Significance of the effect of the P90A substitution was evaluated for each of the four mutants by paired ratio T test in GraphPad Prism.

Yamashita and coworkers previously reported that the HIV-1 mutants E45A and Q63A/Q67A, which are impaired for infection of nondividing cells, can be rescued by addition of CsA [2]. These observations suggested that nuclear entry of these mutants is inhibited by binding of CypA to the mutant capsids. We confirmed their results and extended the analysis to two additional mutants with impaired nuclear entry: E180A and E212A/E213A [12]. We previously showed that viral cores purified from each of these mutants exhibited increased breakage upon strong physical compression in vitro, indicative of reduced elasticity. As previously observed, infection of Hela cells by each of these mutants was significantly reduced by pre-treatment of the cells with aphidicolin, which causes cell cycle arrest at the G1/S phase (Fig 1A). We also observed that addition of CsA markedly increased infection by each of the mutants (Fig 1B & 1C), particularly in arrested cells. Put another way, cell cycle arrest inhibited infection of mutants containing inelastic capsids, and this effect was suppressed by the addition of CsA (Fig 1D).

To confirm that CypA preferentially inhibits infection of arrested cells by the mutants, we analyzed the infection of dividing and arrested Hela cells ablated for CypA expression. Comparing the ratio of infection of dividing vs. arrested cells for each virus, we observed a significant reduction in infection of nondividing cells for each of the four mutants (E45A, Q63A/Q67A, E180A, and E212A/E213A) vs. the wild type (Fig 1D). By contrast, in CypA knockout cells, a marked rescue of infection of nondividing cells was observed for each mutant virus, resulting in no significant difference in cell-cycle dependence of infection vs. the wild type (Fig 1E). Importantly, infection by the two pseudorevertants E45A/R132T and Q63A/Q67E behaved like the wild type, in that no sensitivity to inhibition by cell cycle arrest was observed in both wild type and CypA knockout cells (Fig 1E). We previously showed that these two mutants exhibit improved elasticity relative to their parental mutants, suggesting that reduced capsid elasticity renders HIV-1 mutants susceptible to inhibition by target cell CypA [12].

To establish that CypA binding to the viral capsid is responsible for inhibition of infection of nondividing cells, we constructed mutants bearing both the elasticity reducing mutations and the P90A substitution, which prevents capsid binding by CypA. The addition of the P90A substitution significantly increased the ability of the inelastic capsid mutants to infect nondividing cells, resulting in a reduced infection ratio in untreated and aphidicolin-treated cells (Fig 1F). Collectively, these results establish that CypA binding to the viral capsid selectively inhibits infection of nondividing cells by these HIV-1 mutants that contain inelastic capsids.

### CypA inhibits nuclear entry of HIV-1 mutants with inelastic capsids

**Fig 2:**
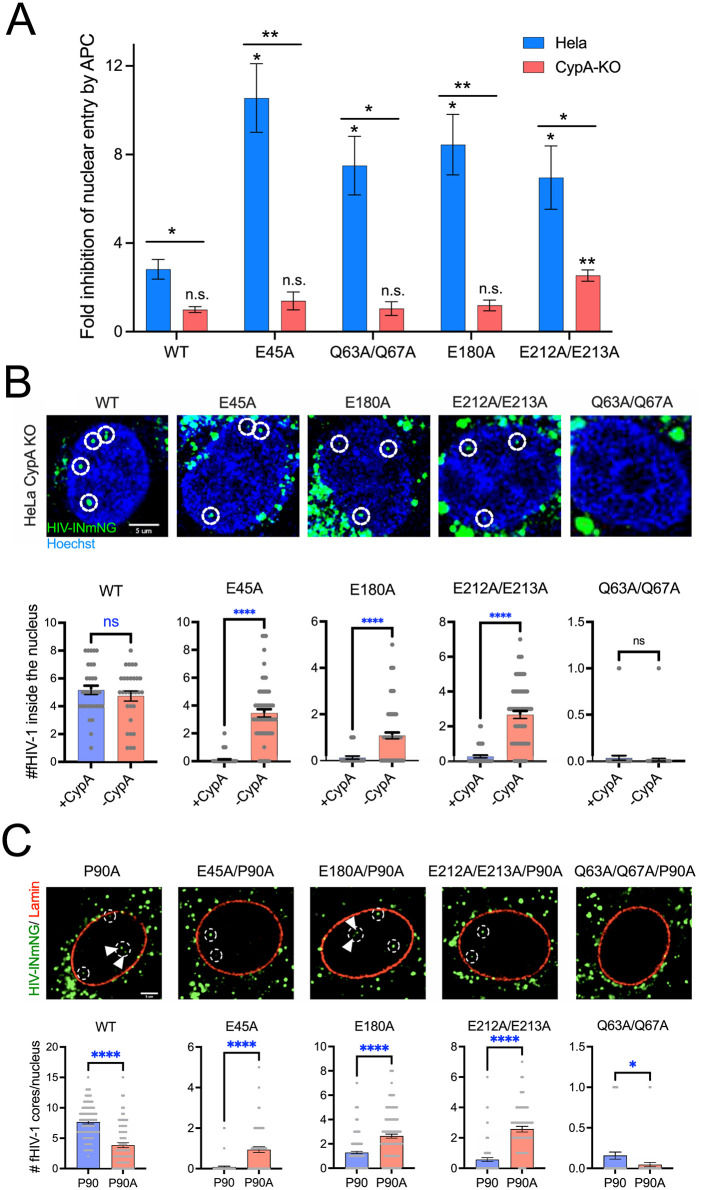
CypA inhibits the nuclear entry of HIV-1 mutants with inelastic capsids. Nuclear entry was monitored in APC-treated Hela cells by (A**)** quantifying the ratio of 2-LTR circles to late reverse transcripts (second strand transfer DNA) by qPCR, or **(**B**)** confocal imaging of INmNG-labeled fluorescently HIV-1 core (fHIV-1) in the nucleus of arrested cells at 8 hours post infection. (C) The nuclear entry of inelastic capsids bearing the P90A substitution was determined in emiRFP-670 tagged lamin-B1 expressing TZM-bl cells. Images of fHIV-1 nuclear entry in aphidicolin-arrested Hela CypA-KO or P90A-mutant infections in WT-TZM-bl cells are shown. Results were compiled from four independent experiments: mean values and standard errors of the mean are shown. Statistical significance was determined using Mann-Whitney Rank Sum test: p > 0.5 not significant (ns); p < 0.0001 (highly significant) is marked by ****. In **(A)**, asterisks placed directly above each bar indicates the significance level relative to the corresponding wild type virus in the indicated cell line. Asterisks above each pair of bars represents analysis of the difference of the indicated virus in Hela vs. CypA KO cells.

To test whether CypA inhibits infection of nondividing cells by preventing HIV-1 nuclear entry, we quantified the accumulation of intracellular HIV-1 DNA synthesized in Hela cells following inoculation with HIV-1. For this purpose, we quantified both nuclear (2-LTR circles) and total (second strand transfer products) reverse transcript forms. Nuclear entry efficiency was computed as the ratio of the former to the latter. Because the CA mutations could also result in variable integration defects, the integrase inhibitor Raltegravir was included to prevent differential elevation of 2-LTR products that could result from mutations also affecting integration. Infections were also performed in arrested cells, and the results were presented as the fold-inhibition of nuclear entry in arrested vs. dividing cells (Fig 2A). In Hela cells, treatment with APC resulted in significantly higher inhibition of nuclear entry of each of the four mutants relative to the the wild type virus. By contrast, inoculation of CypA-deficient cells revealed reduced inhibition of nuclear entry by the mutants in arrested vs. control cells (Fig 2A). Thus, CypA inhibited the nuclear entry of the four inelastic capsid mutants in nondividing Hela cells. We also note that the wild type virus exhibited a 2.9-fold reduction in nuclear entry in Hela cells that was also not observed in CypA-deficient cells. Analysis of the late reverse transcripts produced in the cells showed a general reduction in the levels of viral DNA synthesis in the APC-treated samples, but this effect was typically less than two-fold and was observed for both the wild type and mutant viruses (S1 Fig).

Using a complementary approach, we also examined nuclear entry by tracking the intracellular location of integrase-neonGreen (INmNG)-labeled HIV-1 cores in arrested cells by confocal microscopy. Nuclear entry of the E45A, E180A, and E212A/E213A mutants was markedly increased in cells lacking CypA (Fig 2B). Unexpectedly, while the results of the 2-LTR circle assays indicated increased nuclear entry of Q63A/Q67A in CypA-deficient cells (Fig 2A), the imaging approach failed to detect nuclear entry by this mutant under any of the conditions (Fig 2B and 2C). We have not established the cause of this discrepancy, but it is possible that the Q63A/Q67A cores exhibit unstable association of the labeled IN protein during reverse transcription. Addition of the P90A substitution markedly enhanced nuclear entry of the E45A, E180A, and E212A/E213A mutants in wild type Hela cells (Fig 2C). Collectively, these results show that binding of CypA to the HIV-1 capsid and not RanBP2/Nup358, which contains a cyclophilin homology domain that also binds to CA, inhibits infection of nondividing cells by preventing nuclear entry of the viral core.

### Binding of CypA to the viral capsid reduces the elasticity of native HIV-1 cores

**Fig 3:**
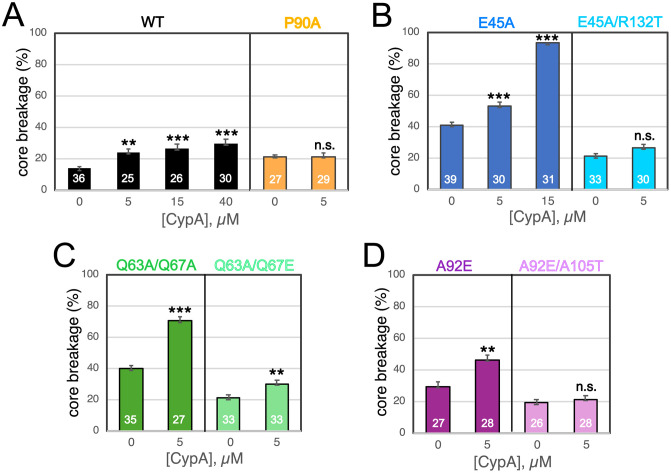
Effects of CypA on the elasticity of purified HIV-1 cores. Viral cores were purified from detergent-treated virions in the presence of IP6 and immobilized on glass slides under native (unfixed and unstained) conditions. Cores were first identified by AFM scanning. A series of cores was then individually subjected to imaging by scanning, compressed by application of the probe at high force, allowed to recover, and rescanned. Broken cores were identified from the images as clearly damaged (broken capsid or altered shape); representative images are provided in S2 Fig. The analysis was subsequently performed on a separate set of attached cores treated with the indicated concentrations of CypA. Results shown are the percentage of cores that underwent breakage upon CypA addition, with error bars depicting the standard error. The number of cores analyzed for each condition is shown within the corresponding bar. **(A)**: wild type and P90A cores; **(B)**: E45A and E45A/R132T cores; **(C)**: Q63A/Q67A and Q63A/Q67E cores; **(D)**: A92E and A92E/A105T cores. The error of the mean was determined via bootstrap analysis. Significance of the effect of CypA (relative to the respective no-CypA value) was evaluated using a Chi-squared test for independence. n.s.: p > 0.05; **: p < 0.01; ***: p < 0.001. Data from measurements of WT, E45A, E45A/R132T, Q63A/Q67A and Q63A/Q67E without CypA were taken from Deshpande et al. [12].

In principle, coating of the viral capsid by CypA could inhibit nuclear entry sterically by increasing the effective size of the core. However, the ability of suppressor mutations distal to the CypA binding site to rescue nuclear entry in nondividing cells suggested the possibility of a different mechanism. We previously showed that the suppressors reversed the reduced elasticity of the original mutants, suggesting that CypA binding may further alter the elasticity of the capsid. To test this, we employed atomic force microscopy imaging to examine the state of HIV-1 cores following strong forced compression (reflecting the elasticity of the capsid). To quantify elasticity, individual viral cores were subjected to compression, and the fraction of cores that underwent permanent structural damage was determined by AFM imaging. Images of several individual cores acquired before and after compression are shown in S2 Fig. Addition of CypA increased the breakage of wild type cores in a concentration-dependent manner, resulting in 30% of cores undergoing breakage at a CypA concentration of 40 μM (Fig 3A). As a control for potential nonspecific effects of the recombinant CypA protein, we also tested its effect on HIV-1 cores bearing the P90A substitution in CA, which abolishes CypA binding. Intriguingly, the mutant cores exhibited approximately 50% increased breakage vs. the wild type in the absence of CypA (Fig 3A). Addition of CypA had no effect on the breakage frequency (Fig 3A), confirming that binding of CypA is required for its effect on the elasticity of HIV-1 cores.

CypA increased the breakage of E45A cores from an initial level of 41% (no CypA) to 53% (5 μM CypA) and 90% (15 μM CypA) (Fig 3B). Upon addition of 15 μM CypA, a large fraction of the cores underwent breakage even prior to AFM compression. Following treatment of immobilized E45A cores with 40 μM CypA, no unbroken cores were observed even in the absence of compression. Images of several broken E45A cores imaged without compression are shown in S3 Fig. Thus, at high CypA concentrations, E45A cores underwent destruction, consistent with a previous study reporting disruption of assembled CA tube structures by 40 μM CypA [19]. Collectively, these results indicate that CypA decreases the elasticity of the E45A mutant HIV-1 capsid, resulting in capsid breakage at high concentrations.

As previously reported [12], cores purified from the E45A pseudorevertant E45A/R132T exhibited a breakage frequency approximately half that of E45A mutant cores (Fig 3B). Addition of CypA to E45A/R132T cores increased their brittleness only slightly, resulting in core breakage similar to CypA-bound wild type cores (Fig 3B).

Addition of CypA also increased the breakage of intrinsically inelastic Q63A/Q67A mutant cores (Fig 3C). By contrast, cores from the pseudorevertant Q63A/Q67E exhibited breakage levels approximately half that of the Q63A/Q67A and were less affected by CypA (Fig 3C). Together, these results indicate that CypA inhibition of nuclear entry is linked to its effects on HIV-1 core elasticity.

In Hela cells, nuclear entry of the CsA-resistant mutant A92E mutant is inhibited by CypA, particularly in arrested cells. This effect is reversed by addition of the suppressor mutation A105T [14,16]. Given the phenotypic similarity between the effects of CypA on infection by inelastic capsid mutants and its effects on CsA-dependent mutants, we evaluated the elasticity of purified A92E mutant cores. In the absence of CypA, approximately 29% of A92E cores underwent breakage upon compression (Fig 3D), suggestive of elasticity between that of wild type and E45A mutant cores. Addition of 5 μM CypA increased the breakage frequency to 46%—roughly double that of wild type cores. By contrast, the elasticity of cores purified from the A92E/A105T pseudorevertant was unaffected by CypA (Fig 3D). These results suggest that the impaired elasticity of the A92E mutant capsid and the further reduction by CypA are functionally related to the impaired ability of the A92E mutant virus to infect nondividing cells.

### The E45A substitution does not detectably alter the affinity of CypA for the viral capsid, while the A92E substitution leads to 2-fold increase in the dissociation constant

**Fig 4:**
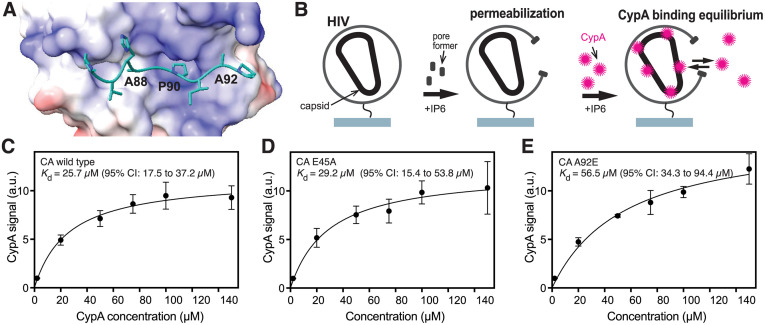
CypA affinity for HIV-1 cores in vitro is not affected by the E45A substitution, but the A92E substitution leads to a 2-fold increase in the dissociation constant. **(A)**: Structural model for the CypA binding loop of HIV-1 CA bound to CypA. **(B)**: Diagram illustrating the experimental approach. Virions were immobilized onto glass, permeabilized with Streptolysin O, and incubated with various concentrations of fluorescently labeled CypA. Binding of CypA to the viral capsid is detected and by TIRF microscopy and quantified by image analysis. **(C-E)**: Median CypA signal (filled circles) bound at equilibrium to cores in permeabilized HIV-1 virions containing wild type **(C)**, E45A **(D)**, and A92E **(E)** CA. CypA binding was measured by TIRF microscopy as a function of CypA concentration and fit of an equilibrium binding model (black line).

The E45 side chain is located at an intersubunit interface in the capsid hexamer, while A92 lies within a flexible loop that is exposed on the outer surface of the capsid lattice and to which CypA binds (model shown in Fig 4A). CypA binding to the HIV-1 capsid results in helical array of protein on the capsid surface, and binding could be cooperative and affected by changes in lattice properties [20,21]. To determine whether changes in capsid-binding affinity may account for the inhibition of nuclear entry by CypA, we tested whether E45A and A92E substitutions affect CypA binding to HIV-1 cores. Virions were immobilized on glass coverslips and permeabilized with the pore-forming protein Streptolysin O followed by addition of fluorescently labeled CypA protein (Fig 4B). Binding of CypA to cores was quantified via TIRF microscopy [22]. Fitting of the binding data measured at a range of CypA concentrations with an equilibrium binding model yielded a dissociation constant (K_d_) of 25.7 μM for cores with wild type CA (Fig 4C). As expected, the K_d_ measured with CA E45A cores (29.2 μM) was comparable to the value for the wild type (Fig 4D). By contrast, the A92E substitution led to a modest decrease in CypA affinity to the core (K_d_ = 56.5 μM; Fig 4E). This 2-fold increase in K_d_ is consistent with the reported effects of other substitutions at A92 on CypA binding measured by surface plasmon resonance (wild type CA, 15 + /-5 μM; CA A92G, 32 μM; CA A92V, 22 μM) [23]. At the estimated CypA concentrations in the cell (10–30 μM), this decrease in affinity would theoretically lead to a reduction in CypA binding to the core of 35–50%. Collectively, our results suggest that inhibition of E45A and A92E infection by CypA is not due to tighter binding of the host protein to the mutant capsids.

### The CA suppressor mutations A105T and R132T rescue infection of nondividing cells by the E45A mutant and by CsA-dependent mutants

**Fig 5:**
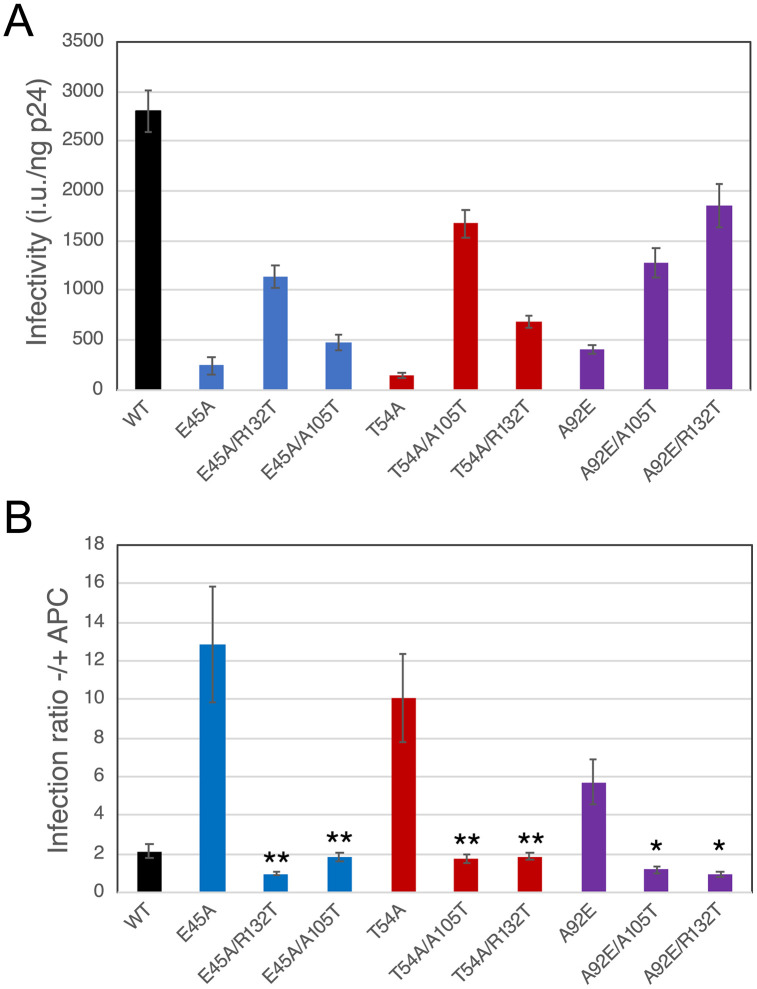
Reciprocal rescue of nondividing cell infection by suppressors of inelastic and CsA-dependent mutants. Infection of dividing and arrested Hela cells by wild type and mutant HIV-GFP reporter viruses was quantified by flow cytometry. **(A)**: Infectivity of the viruses on dividing cells, normalized by the p24 content of the respective inocula. **(B)**: Ratio of infection on dividing and arrested cells. Asterisks shown in panel B indicate significant differences between the infection ratio for each double point mutant relative to the corresponding single mutant. Shown are the mean values of results from three independent experiments with error bars representing standard error values. Significance was determined by paired ratio T test comparing each double mutant relative to its corresponding single mutant.

Our results reveal a phenotypic similarity between the inelastic capsid mutants E45A, Q63A/Q67A, E180A, and E212A/E213A and the CsA-dependent mutants T54A, A92E, and G94D. Because the E45A and the A92E infection phenotypes and CypA-dependent elasticity impairments were rescued by distinct suppressor mutations, we asked whether the suppressors could act reciprocally to rescue infection of arrested Hela cells. To this end, we created mutants bearing the reciprocal suppressors (E45A/A105T, T54A/R132T, and A92E/R132T) and assayed them for infection of arrested cells. We observed enhanced infectivity of each double mutant relative to its parent (Fig 5A). In arrested cells, the mutants also exhibited improved infection relative to their parental viruses, such that the overall inhibition by aphidicolin treatment was significantly reduced by the suppressors (Fig 5B). These results demonstrate that the CsA-dependent mutant suppressor A105T increases the infectivity of the inelastic capsid mutant E45A and reverses its inability to infect nondividing cells. Reciprocally, the E45A suppressor mutation R132T rescued infection of nondividing cells by the CsA-dependent mutants T54A and A92E. These results demonstrate that suppressor mutations that reverse capsid elasticity defects also restore the ability of HIV-1 mutants to infect nondividing cells.

### Analysis of CypA inhibition of infection by CA mutants in T cell lines

Although it is well established that the infection phenotype of the CsA-dependent mutant A92E varies among human cell lines [24–27], the CsA dependence of the nuclear entry-defective mutants E45A, Q63A/Q67A, E180A, and E212A/E213A has not been examined in T cells. Therefore, we analyzed these viruses in single cell infection assays in five T cell lines: CEM, H9, Jurkat, MT-4, and SupT1. For comparative purposes, we included the CsA-dependent CA mutants T54A and A92E and the CsA-independent double mutants T54A/A105T and A92E/A105T. To discern whether CypA inhibits infection, cells were inoculated with the viruses in the presence and absence of CsA. As previously reported, CsA markedly stimulated infection by A92E in H9 cells and, to a lesser extent, in CEM-SS cells (S4 Fig). As expected, this effect was suppressed by the A105T mutation. Infection by the A92E mutant was not stimulated by CsA in SupT1, Jurkat, and MT-4 cells. A similar pattern was observed for the T54A mutant. Infection by the E45A mutant was also stimulated by CsA in H9 cells, and this was reversed by inclusion of the R132T suppressor mutation. Infection by the E180A mutant was stimulated by CsA in H9 CEM, and MT-4 cells. By contrast, the Q63A/Q67A and E212A/E213A mutants were not stimulated by CsA in any of the T cell lines. Collectively, these results reveal cell type differences in CypA restriction of infection HIV-1 mutants with reduced capsid elasticity similar to those observed with other CsA-dependent mutants.

## Discussion

In this study, we show that CypA, a protein that binds to the HIV-1 capsid in the cytoplasm, can inhibit infection of nondividing cells by preventing nuclear entry. Through the analysis of HIV-1 mutants bearing substitutions in the capsid protein, we linked the inhibition of nuclear entry by CypA to its effect on the elasticity of the viral capsid. For mutants with intrinsically brittle capsids (E45A and Q63A/Q67A), CypA binding further reduced their elasticity in an additive manner. Suppressor mutations reversed the effects of CypA on elasticity and restored infection of nondividing cells. We conclude that binding of CypA to the viral capsid inhibits the ability of HIV-1 mutants to infect nondividing cells by reducing capsid elasticity.

For the four mutants previously reported to contain inelastic capsids (E45A, Q63A/Q67A, E180A, and E212A/E213A), CypA inhibited nuclear entry. Cores from the A92E mutant virus exhibited an intermediate level of elasticity that was also reduced by CypA. A92E and several other CsA-dependent mutants are impaired for infection of nondividing cells [17,28], but reports regarding their competency for nuclear entry have been conflicting, possibly owing to heavy reliance on PCR assays for 2-LTR circle forms to analyze HIV-1 nuclear entry [17,28,29]. CypA has also been shown to alter HIV-1 integration targeting [30,31] and preintegration complex function [32], suggesting that the host protein also affects viral capsid function in the nucleus. Collectively, our results suggest that a threshold level of capsid elasticity is required for efficient HIV-1 nuclear entry.

Recent groundbreaking studies have observed intact or nearly intact HIV-1 cores within the cell nucleus, challenging an earlier view that disassembly of the viral capsid must occur in the cytoplasm to permit nuclear entry [9,33,34]. Moreover, CypA bound to the viral capsid in the cytoplasm apparently dissociated from the core prior to or during passage through the nuclear pore [34]. Our results are compatible with this view. While we observed that the inhibition of nuclear entry by CypA was most pronounced for mutants with intrinsically inelastic capsids, we also detected a small reduction in nuclear entry of wild type HIV-1 and a reduction in wild type core elasticity. These observations, coupled with our finding that CypA binding reduced the elasticity of mutant HIV-1 cores, suggest that CypA regulates either the timing or efficiency of nuclear entry by modulating the elasticity of the capsid. Accordingly, CypA was previously observed to slow the nuclear entry of wild type HIV-1 using cell imaging [35].

The inhibitory effect of CypA on HIV-1 infection varies substantially with the cell type used for infection. Our study employed Hela cells that are frequently used in studies of HIV-1 infection. The cell-type variation of the CsA-dependent mutant phenotype has been linked to a dominant activity in Hela cells, suggesting the existence of a cellular factor that inhibits infection in cooperation with CypA [36]. A plausible candidate is the capsid-binding protein CPSF6, which restricts infection by the E45A, Q63A/Q67A, A92E, G94D, and T54A mutants in a CypA-dependent manner. Moreover, some CA substitutions that reduce CPSF6 binding to the viral capsid, including N74D and A105T, were also found to suppress the CsA-dependent phenotype [37,38]. However, the cell type dependence of the phenotype was not linked to differential expression of CPSF6 [27], so a third host factor may also be involved. The inner nuclear envelope proteins Sun1 and Sun2 are candidates, as both bind CA nanotubes and inhibit HIV-1 nuclear entry [39]. Sun2 contributes to the antiviral effect of CypA on specific HIV-1 and HIV-2 mutants [40]. However, its contribution to inhibition of other CsA-dependent mutants has not been demonstrated. Overexpression of Sun1 inhibits nuclear entry of wild type HIV-1 by a CypA-dependent mechanism [41]. It will be of interest to determine whether these proteins bind directly to the HIV-1 capsid, and if so, to test their effects on the elasticity of HIV-1 cores.

We also observed that the R132T suppressor of E45A rescued infection of arrested cells by the CsA-dependent mutants A92E and T54A; reciprocally, the A105T suppressor of CsA-dependent mutants rescued E45A infection of arrested cells. These mutations restored elasticity to their parent mutants [12], suggesting that HIV-1 adapts to drug selection and host cell pressure by passing through fitness troughs involving changes in capsid properties, possibly including elasticity. We previously showed that the capsid-binding inhibitors PF74 and Lenacapavir reduce HIV-1 capsid elasticity at concentrations of the compounds that inhibit nuclear entry of the viral core [12]. Inhibition of HIV-1 infection by PF74 is enhanced by binding of CypA to the viral capsid [42], an effect that may be related to CypA’s effect on elasticity. We suggest that HIV-1 may evolve resistance to capsid inhibitors through pathways that alter capsid elasticity. Since elasticity appears to be critical for nuclear entry and CPSF6 binding is preserved in vivo [37], the resulting fitness costs of changes that affect either may limit the emergence of resistance to capsid inhibitors in vivo.

Our results further demonstrate the importance of capsid elasticity in HIV-1 nuclear entry and establish that it is controlled by the binding of a host protein. However, we have not yet identified the mechanism by which core elasticity promotes traversal of the NPC. One possibility involves compression exerted on the core during penetration of the nuclear pore owing to the presence of the high density of nucleoporin side chains in the central plug. Supporting this hypothesis is the observation that the NPC can undergo structural damage (termed “cracking”) during HIV-1 nuclear entry, suggesting that strain may be experienced by the core during penetration of the pore [33,34]. The dynamic interactions of the capsid with FG repeat polypeptides occurring during nuclear pore penetration may further alter the properties of the capsid in a manner that is not yet understood. Finally, capsid-binding host proteins that inhibit HIV-1 nuclear entry, including Mx2, CPSF6–358, and TRIM5a, may do so by modulating capsid elasticity.

While our results link CypA’s effect on elasticity to its ability to inhibit nuclear entry of HIV-1 mutants, this action may be distinct from the beneficial effect of CypA in wild type HIV-1 infection, which has been attributed to protection of the viral core from restriction by TRIM5a and cloaking of the reverse transcribed viral DNA from cytoplasmic sensing mechanisms [43–45]. The apparent shedding of CypA from the capsid during nuclear entry could resolve this dilemma [34]. Thus, it appears that the HIV-1 capsid has evolved to engage the host cell protein CypA for protection in the cytoplasm while evading its ability to inhibit nuclear entry. These opposing demands may have contributed to the high sensitivity of the multifunctional HIV-1 capsid protein to mutations [46].

## Online methods

### Cells and viruses

Hela cells were purchased from the American Type Culture Collection. The clonal cell line Hela-CypA KO was generated by CRISPR knockout of the cyclophilin A gene in Hela cells using the guide sequence cloned into pLenti-CRISPR2, as previously described [47,48]. Depletion of endogenous CypA was demonstrated by immunoblotting of cell lysates. Cells were cultured in DMEM containing 10% fetal bovine serum and penicillin and streptomycin. Aphidicolin and cyclosporin A were purchased from Sigma and dissolved in water and DMSO, respectively. Solutions were stored in aliquots at -80°C and thawed prior to use.

The HIV-GFP reporter viral proviral plasmids encoding substitutions in CA were previously described: E45A, E45A/R132T, Q63A/Q67A, Q63A/Q67E, E180A, E212A/E213A [12]. HIV-GFP plasmids encoding E45A/A105T, T54A, T54A/A105T, T54A/R132T, A92E/A105T, and A92E/R132T were constructed by transferring BssHII-SpeI, BssHII-ApaI, and SpeI-ApaI restriction fragments from the corresponding R9 molecular clones, based on the location of the mutations in the HIV-1 genome [16,17].

Viruses were generated by transfection of 293T cell monolayers in 10 cm dishes using polyethyleneimine [49]. Supernatants were harvested 36-42h post-transfection, clarified by centrifugation, and passed through 0.45 μM syringe filters. Aliquots were frozen and stored at -80°C until used for infection assays. Infections of Hela cells were performed in 24-well plates, as previously described [12]. Extent of infection was determined by flow cytometry for GFP expression in cell suspensions that had been inactivated by chemical fixation. For infections of arrested cells, cells were seeded in culture media containing aphidicolin (2 μg/ml). The virus stocks were diluted in media containing aphidicolin. One day after inoculation, the inoculum was removed by aspiration and the cultures replenished with fresh medium lacking aphidicolin. In experiments involving CsA, the drug was added to the cell culture media to a final concentration of 5 μg/ml.

To measure viral infectivity, viral titers were calculated from the dilution used in the infection and the percentage of infected cells and divided by the p24 concentration of the inoculum. The latter was determined using an in-house ELISA protocol, as previously described [50].

Nuclear entry was assayed by qPCR quantification of 2-LTR circles and late reverse transcripts in purified total cellular DNA, as previously described [12].

### Expression and purification of recombinant HisCypA

N-terminally His-tagged CypA (HisCypA) was recombinantly expressed in E. coli strain C41 (DE3). Cultures were grown in 2 × YT medium with ampicillin and induced mid-log phase with 1 mM IPTG for overnight expression at 18°C. Cells were pelleted, resuspended in buffer (50 mM Tris, pH = 9.0; 150 mM NaCl; 1 mM DTT; cOmplete protease inhibitor; 20% BugBuster (Merck Millipore)), and lysed by sonication. Lysates were clarified, and His-tagged protein was captured by immobilized metal ion affinity chromatography on a 5 mL Ni2 + -NTA HP column (Cytiva). Following a wash step (50 mM Tris pH 9.0, 150 mM NaCl, 1 mM DTT, 20 mM imidazole), HisCypA was eluted using buffer containing 300 mM imidazole. To remove nucleic acids, the eluate was passed through a 5 mL Q anion exchange column (Cytiva). Final purification was performed via size exclusion chromatography on a HiLoad Superdex 75 pg column (Cytiva) with the final storage buffer (50 mM Tris, pH 9.0; 150 mM NaCl; 1 mM TCEP).

### Quantification of HIV-1 nuclear entry by confocal microscopy

Nuclear entry of HIV-1 capsid mutants was determined as previously described [51,52]. In brief, 8 × 10^4^ or 5 × 10^5^ TZM-bl cells that stably express the emiRFP670-laminB1 nuclear envelope marker, or the Hela parental (control) and CypA-knock-out (CypA KO) cells, were plated in a 8-well chambered slide (#C8-1.5H-N, CellVis) for single time-point nuclear import experiments. Aphidicolin was added to cells (10 µM final concentration) to block cell division. About 14h later, the cells were infected with wild type and mutant HIVeGFP particles fluorescently tagged with Vpr-integrase-mNeongreen (INmNG) to label the viral core. Inoculum concentrations were determined based on an MOI of one for the wild type and equivalent particle concentrations of the mutant viruses as normalized by reverse transcriptase activity in viral lysates. Virus binding to cells was augmented by spinoculation (1500 × g for 30 min, 16°C), and virus entry was synchronously initiated by adding pre-warmed complete DMEM medium containing aphidicolin (10 µM) to samples mounted on a temperature- and CO_2_-controlled microscope stage. 3D confocal imaging was carried out on a Leica SP8 LSCM using a C-Apo 63x/1.4NA oil-immersion objective. Tile-scanning was employed to image multiple (4x4) fields of view. For assessing HIV-1 nuclear entry in a fixed time-point (8 hpi), stringent imaging conditions were used, i.e., 4x line-averaging, with 0.12 μm/pixel sizes and 0.5 μm spaced z-stacks. 488 and 633 nm laser lines was used to excite the INmNG and emiRFP670-Lamin B1 fluorescent markers, and their respective emission was collected between 502–560 nm and 645–700 nm using GaSP-HyD detectors. For Hela cell experiments, the nuclei were labeled with Hoechst dye and imaged using 405 nm lasers, and emission was detected using GaSP-HyD detectors set at 420–480 nm. 3D-image series were processed off-line using ICY image analysis software (http://icy.bioimageanalysis.org/) [53]. HIV-1 nuclear entry in fixed time-point 3D z-stack images was analyzed using an in-house script in the ICY protocols’ module as described in [54].

### AFM assay of HIV-1 core elasticity

Samples for AFM measurements and analysis were prepared as previously described [55]. Briefly, 10 µL of isolated HIV-1 cores were incubated for 45 min at room temperature on hexamethyldisilazane (HMDS)-coated microscope glass slides (Sigma-Aldrich) in a mildly humidified chamber, thereby preventing evaporation from the droplet. AFM measurements were performed on the adhered sample without fixation. Each experiment was repeated at least three times, each time with independently purified pseudoviruses. IP6 was purchased from Sigma-Aldrich (P8810). All measurements were carried out with a JPK Nanowizard Ultra-Speed atomic force microscope (JPK Instruments, Berlin, Germany) mounted on an inverted optical microscope (Axio Observer; Carl Zeiss, Heidelberg, Germany). Silicon nitride probes (mean cantilever spring constant of 0.12 N/m; DNP, Bruker, Germany) were used. Topographic images were acquired using the quantitative imaging (QI) mode at a rate of 0.5 lines/s and a loading force of 300 pN and rendered using the WSxM software (Nanotec Electronica).

Core elasticity was assessed as previously described [12,56]. First, using a low loading force (300 pN), we scanned a region (350 × 350 nm; 128x128 pixels) that covered the entire core. Next, a small rectangular region covering the central section of the core (350 nm X 50 nm; 64x11 pixels) was scanned at 300 pN loading force to determine the initial volume underneath the region. Compression was then applied by rescanning the same region at a loading force of 5 nN, after which the force was reduced back to 300 pN. Following compression, the entire core was subsequently reimaged at 300 pN to evaluate structural alterations. Structural integrity of the core was determined by comparing full pre- and post-compression images, and elasticity was quantified as the percentage of intact versus broken cores following forced compression.

### TIRF microscopy assay of CypA binding

HIV particles were produced in HEK-293T cells transfected using PEI with a mixture of the plasmids pCRV1-GagPol and pCSGW. The medium was exchanged 18 hr post transfection and the virus-containing medium was collected 66 hr post transfection and centrifuged (2100 x *g*, 20 min, 4 °C) to remove cellular debris. The supernatant was concentrated using a 100 kDa MWCO centrifugal filter. HIV particles were purified using a Sephacryl S500 size exclusion column and labelled with biotin using NHS ester chemistry (ThermoFisher, EZ-Link NHS-LC-LC-Biotin, #21343). The biotinylated HIV were purified using a Sephacryl S500 size exclusion column, concentrated and flash-frozen in liquid nitrogen. TIRF microscopy binding assays were performed using microfluidic channel devices as described previously [PMID: 29877795]. HIV-1 particles were immobilized on PLL-PEG-biotin–modified coverslips, permeabilized in the presence of 0.1 mM IP6 with the pore-forming protein streptolysin O and incubated for 30 min to allow disassembly of improperly assembled cores (which cannot be stabilized by IP6). A solution containing 2 µM AF647-labelled CypA (produced and labelled as described [47]) was mixed with varying amounts of unlabelled CypA to yield final CypA concentrations of 2–150 µM and introduced into the flow channel. CypA binding was quantified by measuring the intensity of diffraction-limited AF647-CypA spots colocalizing with HIV-1 particles in TIRF images. A full concentration titration was performed within each flow channel, and each dataset represents at least three independent channels. Image analysis was carried out using software generated in house (https://github.com/lilbutsa/JIM-Immobilized-Microscopy-SuitE).

## Supporting information

S1 Fig Analysis of reverse transcription of wild type HIV-1 and CA mutants in Hela cells expressing and lacking CypA. Second strand transfer products were quantified by qPCR in total DNA purified from wild type (left panel) and CypA KO Hela cells (right panel) following inoculation with equivalent quantities (normalized by RT activity) of VSV-G-pseudotyped HIV-GFP reporter viruses. Values are the ratio of late product synthesis in dividing to arrested (APC-treated) cells. Each value was normalized by the corresponding concentration of total DNA in the cell extracts. Shown are results from two experiments. Data are provided in S1 Data file (Fig 2A tab).
(TIFF)

S2 Fig Topographic AFM images of isolated IP6-treated cores before and after high-force (5 nN) compression. All images were acquired using the Quantitative Imaging (QI) mode at a loading force of 300 pN. For clarity, openings or damages in the cores are shown within a dashed yellow rectangle. Selected cores are shown (WT and mutants) and are grouped based on whether they were broken upon compression. Scale bars are 50 nm.
(TIFF)

S3 Fig Topographic AFM images of isolated IP6-treated E45A mutant cores before and after addition of CypA (40 μM final concentration). Images were obtained by AFM in the QI mode at a loading force of 300 pN. For clarity, damaged regions of the cores are shown within dashed yellow rectangles. The observed damage occurred spontaneously following addition of CypA without application of high-force compression. Scale bars represent 50 nm.
(TIFF)

S4 Fig Effect of CsA on infection by wild type and mutant HIV-1 in five human T cell lines. Cells were cultured in RPMI1640 medium supplemented with 10% fetal bovine serum, penicillin, and streptomycin at 37°C in a humidified incubator with 5% CO_2_. For infection assays, 50,000 cells were seeded in 0.2 ml medium in 96 well plates. Cultures were inoculated with reporter viruses in the presence and absence of CsA (5 μM), incubated for 48h, and harvested and fixed overnight in PBS containing 4% paraformaldehyde prior to flow cytometric analysis for GFP expression. Infection was performed using quantities of VSV-pseudotyped HIV-GFP reporter viruses selected to result in readily measurable but not saturated levels of infection. Values shown are the ratio of infection observed in the absence of CsA to that observed in the presence of CsA. Results from two independent assays of duplicate infections are shown. Infection data are provided in S1 Data file (S4 Fig tab).
(TIFF)

S1 Data Excel file containing complete quantitative data from which Figs 1–5, S1, and S4 were derived. (XLSX)
