# Supplementary figures and images for: The host protein cyclophilin A restricts nuclear entry of HIV-1 mutants by reducing the elasticity of the viral capsid
Source: PLoS Pathog. 2026 Jan 29;22(1):e1013910. doi: 10.1371/journal.ppat.1013910 (PMC12871957; doi:10.1371/journal.ppat.1013910)

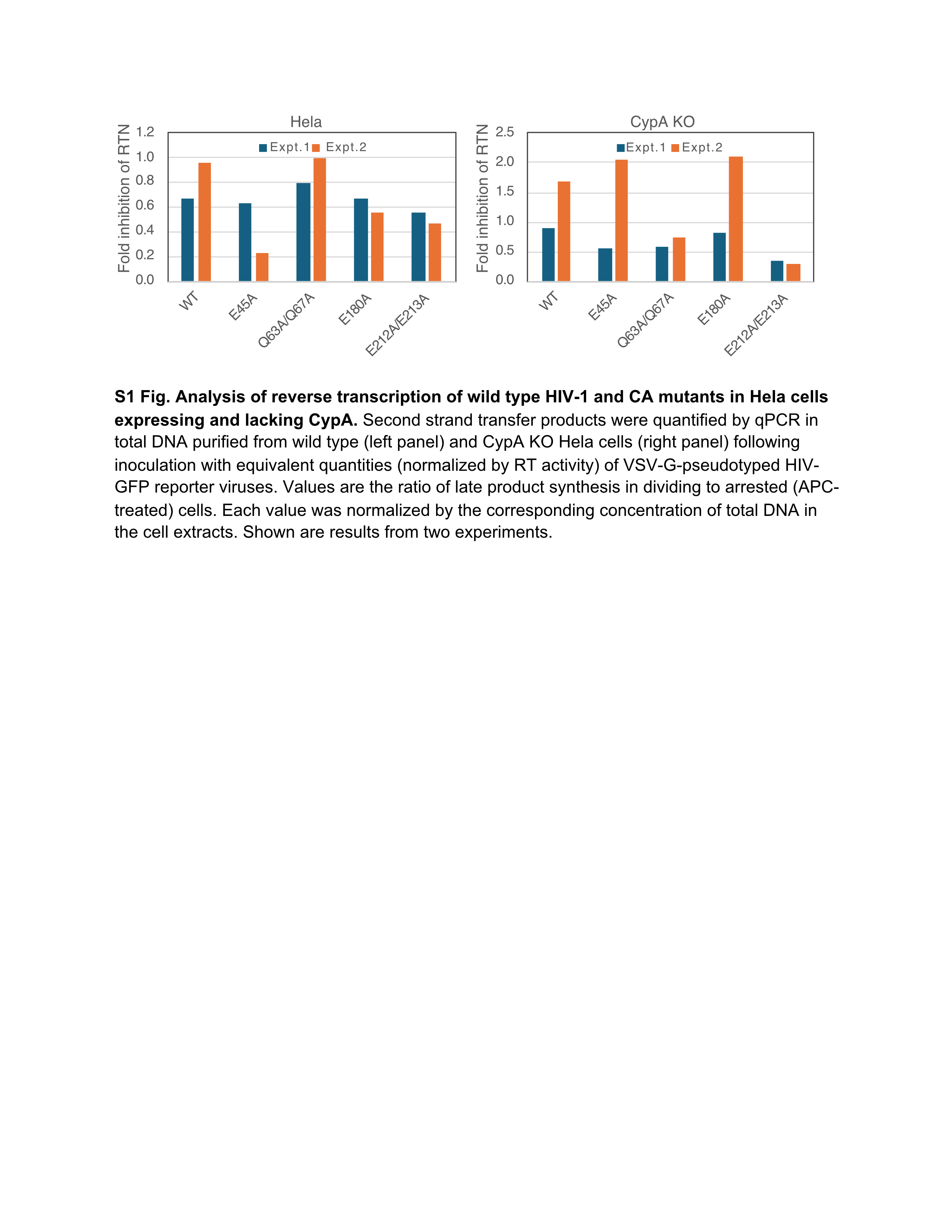

Supplement: S1 Fig — Second strand transfer products were quantified by qPCR in total DNA purified from wild type (left panel) and CypA KO Hela cells (right panel) following inoculation with equivalent quantities (normalized by RT activity) of VSV-G-pseudotyped HIV-GFP reporter viruses. Values are the ratio of late product synthesis in dividing to arrested (APC-treated) cells. Each value was normalized by the corresponding concentration of total DNA in the cell extracts. Shown are results from two experiments. Data are provided in S1 Data file (Fig 2A tab). (TIFF) [file ppat.1013910.s001.tiff]

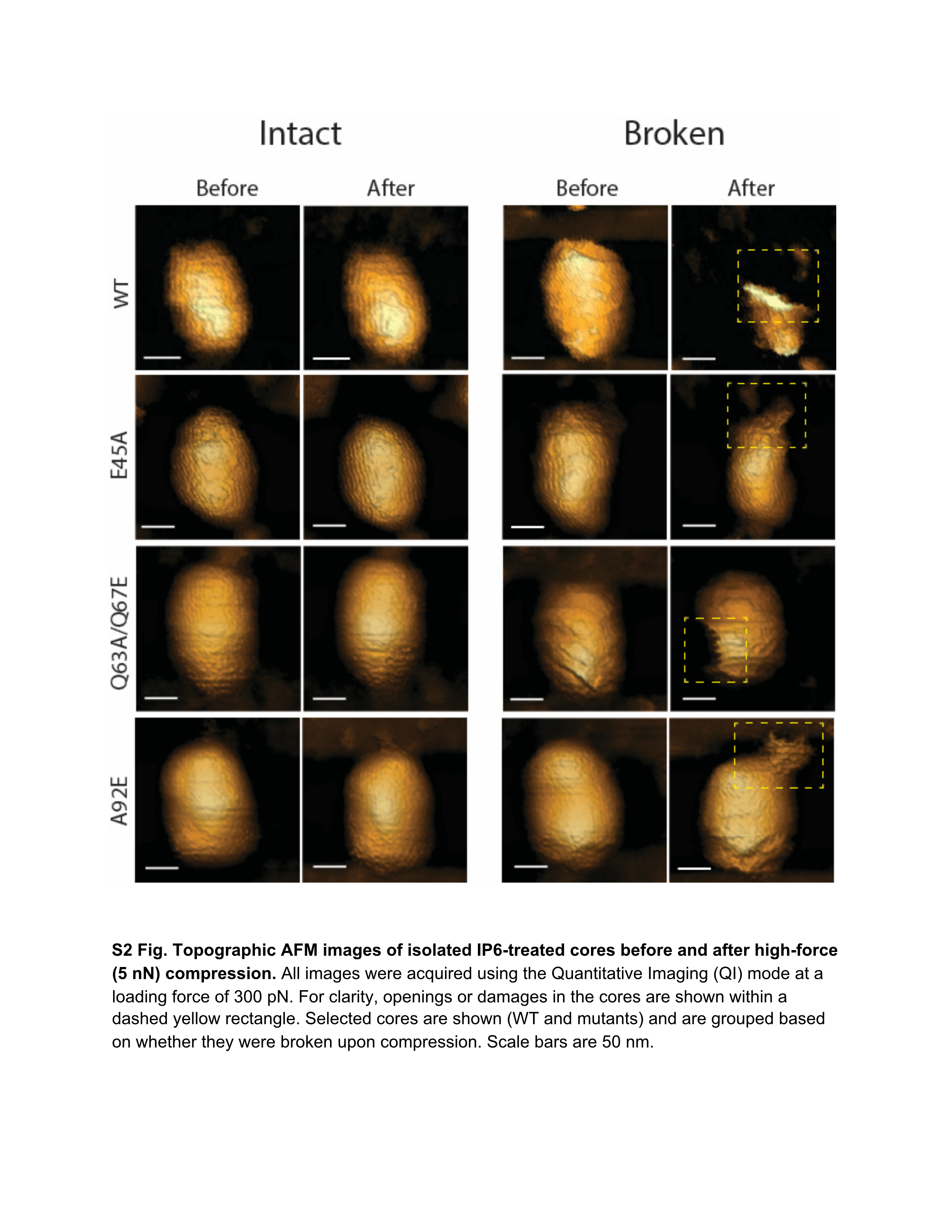

Supplement: S2 Fig — All images were acquired using the Quantitative Imaging (QI) mode at a loading force of 300 pN. For clarity, openings or damages in the cores are shown within a dashed yellow rectangle. Selected cores are shown (WT and mutants) and are grouped based on whether they were broken upon compression. Scale bars are 50 nm. (TIFF) [file ppat.1013910.s002.tiff]

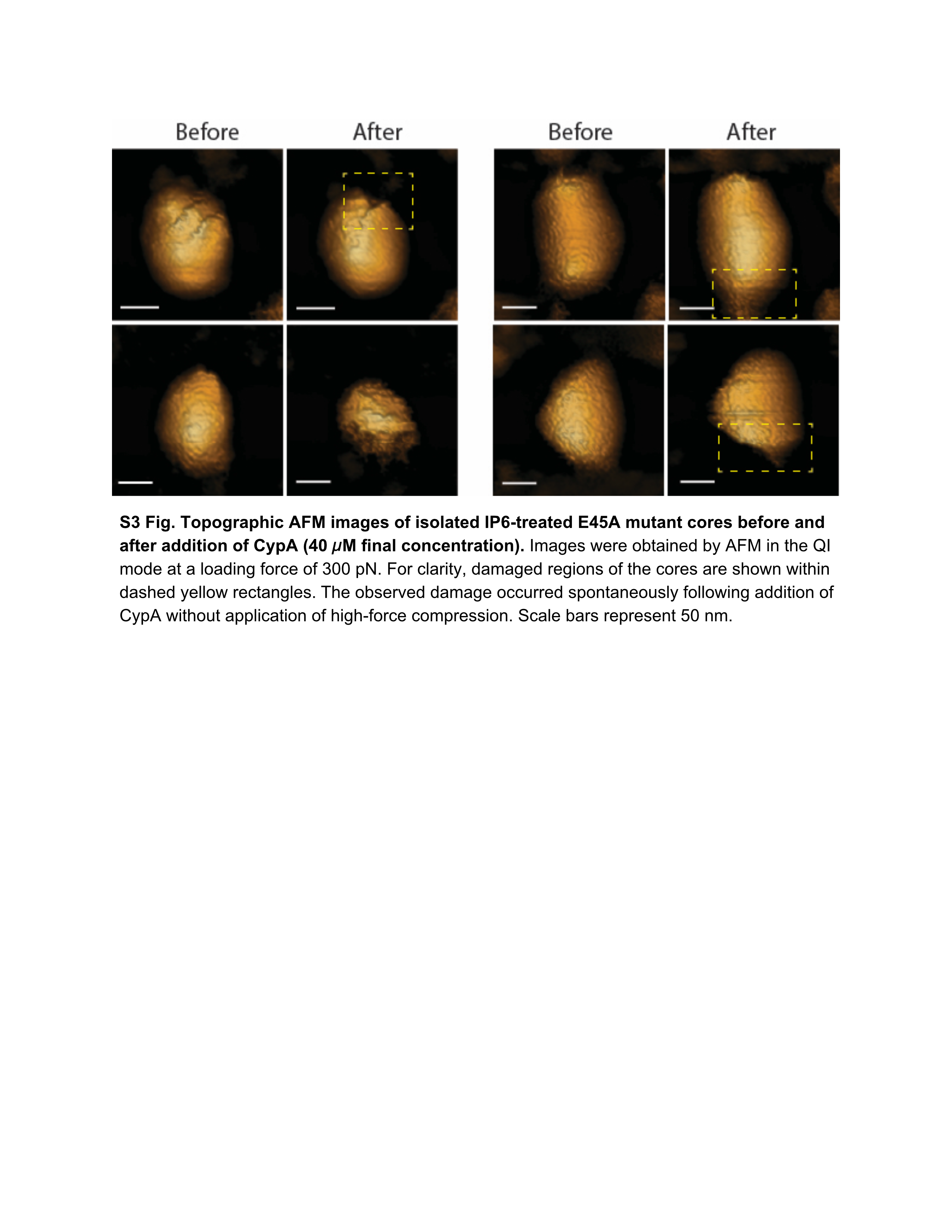

Supplement: S3 Fig — Images were obtained by AFM in the QI mode at a loading force of 300 pN. For clarity, damaged regions of the cores are shown within dashed yellow rectangles. The observed damage occurred spontaneously following addition of CypA without application of high-force compression. Scale bars represent 50 nm. (TIFF) [file ppat.1013910.s003.tiff]

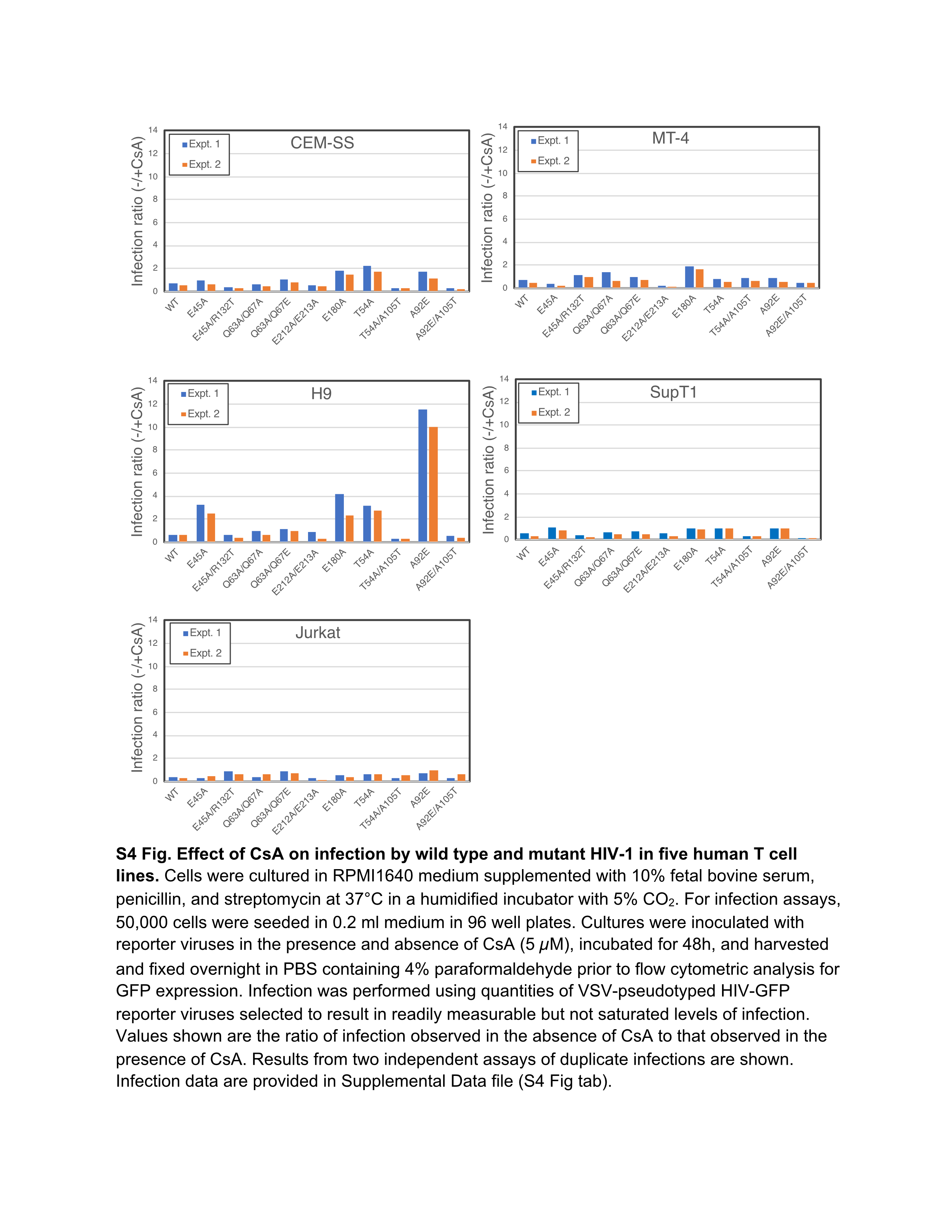

Supplement: S4 Fig — Cells were cultured in RPMI1640 medium supplemented with 10% fetal bovine serum, penicillin, and streptomycin at 37°C in a humidified incubator with 5% CO2. For infection assays, 50,000 cells were seeded in 0.2 ml medium in 96 well plates. Cultures were inoculated with reporter viruses in the presence and absence of CsA (5 μM), incubated for 48h, and harvested and fixed overnight in PBS containing 4% paraformaldehyde prior to flow cytometric analysis for GFP expression. Infection was performed using quantities of VSV-pseudotyped HIV-GFP reporter viruses selected to result in readily measurable but not saturated levels of infection. Values shown are the ratio of infection observed in the absence of CsA to that observed in the presence of CsA. Results from two independent assays of duplicate infections are shown. Infection data are provided in S1 Data file (S4 Fig tab). (TIFF) [file ppat.1013910.s004.tiff]
